# 2-(1-Benzo­thio­phen-2-yl)-4*H*-1,3,4-oxa­diazin-5(6*H*)-one

**DOI:** 10.1107/S1600536813033291

**Published:** 2013-12-14

**Authors:** Hong-Shun Sun, Yu-Long Li, Ning Xu, Lin-Jiang Shen

**Affiliations:** a Chemical Engineering Department, Nanjing College of Chemical Technology, Nanjing 210048, People’s Republic of China; b College of Science, Nanjing University of Technology, Nanjing 210009, People’s Republic of China

## Abstract

In the title compound, C_11_H_8_N_2_O_2_S, the oxadiazinone ring is nearly planar [maximum deviation = 0.016 (4) Å], and is approximately coplanar with the benzo­thio­phene ring system [dihedral angle = 3.1 (5)°]. In the crystal, mol­ecules are linked by N—H⋯O hydrogen bonds, forming chains running along the *b*-axis direction.

## Related literature   

For applications of oxadiazin derivatives, see: De Sarro *et al.* (2005 ▶); Shigeki *et al.* (2012 ▶).
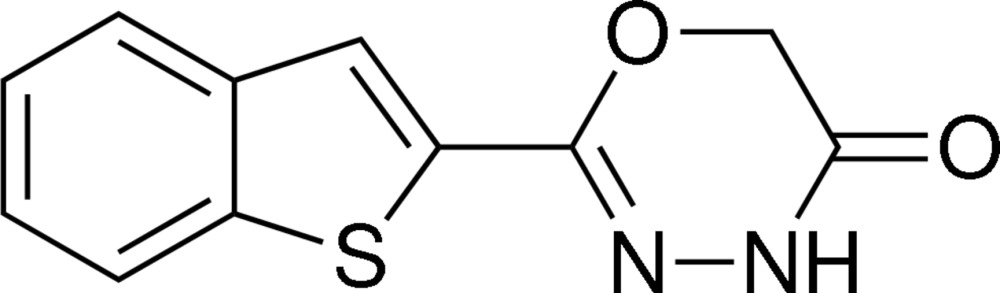


## Experimental   

### 

#### Crystal data  

C_11_H_8_N_2_O_2_S
*M* *_r_* = 232.25
Monoclinic, 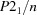

*a* = 7.4950 (15) Å
*b* = 6.0350 (12) Å
*c* = 22.412 (5) Å
β = 93.08 (3)°
*V* = 1012.3 (4) Å^3^
*Z* = 4
Mo *K*α radiation
μ = 0.30 mm^−1^
*T* = 293 K
0.30 × 0.20 × 0.10 mm

#### Data collection  

Enraf–Nonius CAD-4 diffractometer
Absorption correction: ψ scan (North *et al.*, 1968 ▶) *T*_min_ = 0.915, *T*_max_ = 0.970
1999 measured reflections
1848 independent reflections
1167 reflections with *I* > 2σ(*I*)
*R*_int_ = 0.064
3 standard reflections every 200 reflections intensity decay: 1%

#### Refinement  

*R*[*F* ^2^ > 2σ(*F* ^2^)] = 0.064
*wR*(*F* ^2^) = 0.192
*S* = 1.00
1848 reflections
145 parameters
1 restraint
H-atom parameters constrained
Δρ_max_ = 0.39 e Å^−3^
Δρ_min_ = −0.39 e Å^−3^

### 

Data collection: *CAD-4 EXPRESS* (Enraf–Nonius, 1994 ▶); cell refinement: *CAD-4 EXPRESS*; data reduction: *XCAD4* (Harms & Wocadlo, 1995 ▶); program(s) used to solve structure: *SHELXTL* (Sheldrick, 2008 ▶); program(s) used to refine structure: *SHELXTL*; molecular graphics: *SHELXTL*; software used to prepare material for publication: *SHELXTL*.

## Supplementary Material

Crystal structure: contains datablock(s) I, New_Global_Publ_Block. DOI: 10.1107/S1600536813033291/xu5757sup1.cif

Structure factors: contains datablock(s) I. DOI: 10.1107/S1600536813033291/xu5757Isup2.hkl

Supporting information file. DOI: 10.1107/S1600536813033291/xu5757Isup3.cml

Additional supporting information:  crystallographic information; 3D view; checkCIF report

## Figures and Tables

**Table 1:** Hydrogen-bond geometry (Å, °)

*D*—H⋯*A*	*D*—H	H⋯*A*	*D*⋯*A*	*D*—H⋯*A*
N1—H1*A*⋯O2^i^	0.86	2.03	2.848 (5)	158

Symmetry code: (i) 
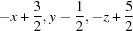
.

**Fig. 1.**
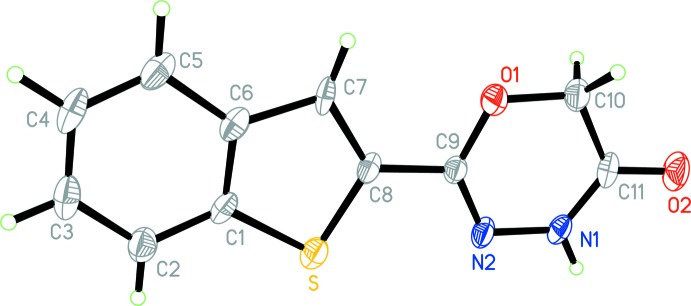
The molecular structure of (I), showing the atom-numbering scheme and displacement ellipsoids at the 30% probability level.

**Fig. 2.**
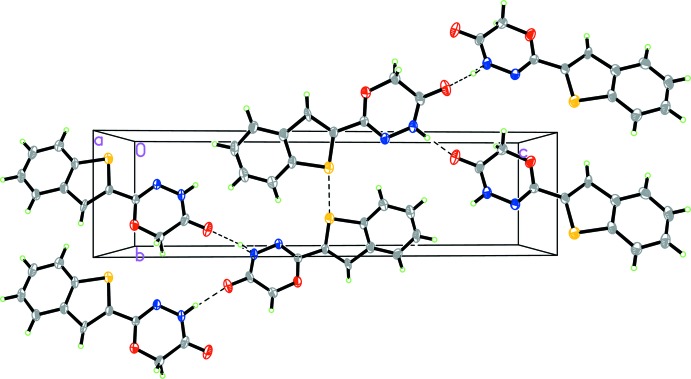
A packing diagram of (I). Intermolecular hydrogen bonds are shown as dashed lines.

## supplementary crystallographic information

### 1. Comment

Noncompetitive α-amino-3-hydroxy-5-methyl-4-isoxazolepropanoic acid (AMPA) receptor antagonists have actively been explored, driven by the belief that such drugs would exercise effectiveness independent of glutamatelevels and the synaptic membrane polarization state, with minor influence on normal glutamatergic activity compared with competitive antagonists (De Sarro *et al.*, 2005). Commercially available 2,4-diphenyl-4*H*-1,3,4-oxadiazin-5-one was selected as a suitable starting compound with a novel structure *versus* other HTS (high throughput screening) hit compounds and known noncompetitive AMPA antagonists (Shigeki *et al.*, 2012). The title compound is a new oxadiazin compound with a similar structure to it. We synthesized it and report here its crystal structure.

The molecular structure of the title compound is shown in Fig. 1. The benzothiophene ring makes a dihedral angle of 3.1 (5)° to oxadiazin ring.

As shown in Figure 2, the molecules are linked by N—H···O and weak S···S (Table 1) bonds into a three-dimensional network, which consolidate the crystal packing.

### 2. Experimental

*N*'-(2-chloroacetyl)benzo[*b*]thiophene-2-carbohydrazide (0.27 g, 1 mmol) was dissolved in 10 ml acetonitrile, after which potassium carbonate (0.28 g, 2.0 mmol) was added and the mixture was refluxed for 3 h under heating. The reaction solution was left and cooled to room temperature, evaporated, diluted with EtOAc, washed with water and brine, and then dried over MgSO_4_. After the drying agent was filtered off, the product was evaporated and the resulting crystalline residues were recrystallized from n-hexane and EtOAc to give the compound (0.19 g, 0.8 mmol, 80.0%) as a yellow crystal suitable for X-ray analysis.

### 3. Refinement

H atoms were positioned geometrically, with N— H = 0.86 Å and C—H = 0.93, and 0.97 Å for aromatic and methylene H, respectively, and constrained to ride on their parent atoms, U_iso_(H) = 1.2U_eq_(C,N).

### Crystal data

**Table:**

C_11_H_8_N_2_O_2_S	*F*(000) = 480
*M**_r_* = 232.25	*D*_x_ = 1.524 Mg m^−^^3^
Monoclinic, *P*2_1_/*n*	Mo *K*α radiation, λ = 0.71073 Å
Hall symbol: -P 2yn	Cell parameters from 25 reflections
*a* = 7.4950 (15) Å	θ = 9–12°
*b* = 6.0350 (12) Å	µ = 0.30 mm^−^^1^
*c* = 22.412 (5) Å	*T* = 293 K
β = 93.08 (3)°	Block, colorless
*V* = 1012.3 (4) Å^3^	0.30 × 0.20 × 0.10 mm
*Z* = 4	

### Data collection

**Table:**

Enraf–Nonius CAD-4 diffractometer	1167 reflections with *I* > 2σ(*I*)
Radiation source: fine-focus sealed tube	*R*_int_ = 0.064
Graphite monochromator	θ_max_ = 25.4°, θ_min_ = 1.8°
ω/2θ scans	*h* = 0→9
Absorption correction: ψ scan (North *et al.*, 1968)	*k* = 0→7
*T*_min_ = 0.915, *T*_max_ = 0.970	*l* = −27→27
1999 measured reflections	3 standard reflections every 200 reflections
1848 independent reflections	intensity decay: 1%

### Refinement

**Table:**

Refinement on *F*^2^	Primary atom site location: structure-invariant direct methods
Least-squares matrix: full	Secondary atom site location: difference Fourier map
*R*[*F*^2^ > 2σ(*F*^2^)] = 0.064	Hydrogen site location: inferred from neighbouring sites
*wR*(*F*^2^) = 0.192	H-atom parameters constrained
*S* = 1.00	*w* = 1/[σ^2^(*F*_o_^2^) + (0.1*P*)^2^ + 0.650*P*] where *P* = (*F*_o_^2^ + 2*F*_c_^2^)/3
1848 reflections	(Δ/σ)_max_ < 0.001
145 parameters	Δρ_max_ = 0.39 e Å^−^^3^
1 restraint	Δρ_min_ = −0.39 e Å^−^^3^

### Special details

**Table:**

Geometry. All e.s.d.'s (except the e.s.d. in the dihedral angle between two l.s. planes) are estimated using the full covariance matrix. The cell e.s.d.'s are taken into account individually in the estimation of e.s.d.'s in distances, angles and torsion angles; correlations between e.s.d.'s in cell parameters are only used when they are defined by crystal symmetry. An approximate (isotropic) treatment of cell e.s.d.'s is used for estimating e.s.d.'s involving l.s. planes.
Refinement. Refinement of *F*^2^ against ALL reflections. The weighted *R*-factor *wR* and goodness of fit *S* are based on *F*^2^, conventional *R*-factors *R* are based on *F*, with *F* set to zero for negative *F*^2^. The threshold expression of *F*^2^ > σ(*F*^2^) is used only for calculating *R*-factors(gt) *etc*. and is not relevant to the choice of reflections for refinement. *R*-factors based on *F*^2^ are statistically about twice as large as those based on *F*, and *R*- factors based on ALL data will be even larger.

### Fractional atomic coordinates and isotropic or equivalent isotropic displacement parameters (Å^2^)

**Table:**

	*x*	*y*	*z*	*U*_iso_*/*U*_eq_	
S	0.66885 (16)	0.2027 (2)	0.99910 (5)	0.0507 (4)	
O2	0.8714 (5)	0.7881 (6)	1.23337 (14)	0.0632 (10)	
O1	0.8545 (5)	0.7527 (5)	1.07326 (14)	0.0606 (10)	
C1	0.6558 (5)	0.2443 (8)	0.92291 (17)	0.0391 (10)	
N1	0.7891 (5)	0.5094 (6)	1.17093 (15)	0.0442 (9)	
H1A	0.7685	0.4264	1.2010	0.053*	
C2	0.5909 (6)	0.0894 (8)	0.8802 (2)	0.0491 (12)	
H2B	0.5496	−0.0488	0.8917	0.059*	
N2	0.7558 (5)	0.4203 (6)	1.11441 (15)	0.0430 (9)	
C3	0.5905 (6)	0.1488 (9)	0.8209 (2)	0.0540 (13)	
H3B	0.5454	0.0508	0.7918	0.065*	
C4	0.6552 (6)	0.3490 (9)	0.8041 (2)	0.0555 (13)	
H4A	0.6566	0.3817	0.7636	0.067*	
C5	0.7186 (6)	0.5054 (8)	0.84494 (19)	0.0514 (12)	
H5A	0.7590	0.6427	0.8325	0.062*	
C6	0.7203 (6)	0.4501 (8)	0.90646 (18)	0.0429 (11)	
C7	0.7809 (5)	0.5961 (8)	0.95690 (16)	0.0371 (10)	
H7A	0.8256	0.7395	0.9550	0.044*	
C8	0.7548 (5)	0.4656 (7)	1.00939 (17)	0.0379 (10)	
C9	0.7923 (5)	0.5458 (7)	1.07104 (17)	0.0342 (9)	
C10	0.8941 (6)	0.8522 (7)	1.13060 (19)	0.0461 (11)	
H10A	0.8284	0.9903	1.1326	0.055*	
H10B	1.0204	0.8878	1.1341	0.055*	
C11	0.8500 (6)	0.7115 (8)	1.18252 (19)	0.0446 (11)	

### Atomic displacement parameters (Å^2^)

**Table:**

	*U*^11^	*U*^22^	*U*^33^	*U*^12^	*U*^13^	*U*^23^
S	0.0665 (8)	0.0529 (7)	0.0334 (7)	−0.0047 (6)	0.0096 (5)	0.0009 (6)
O2	0.076 (2)	0.082 (3)	0.0322 (18)	−0.002 (2)	0.0036 (15)	−0.0179 (18)
O1	0.104 (3)	0.049 (2)	0.0293 (18)	−0.0176 (18)	0.0092 (17)	−0.0028 (14)
C1	0.038 (2)	0.056 (3)	0.024 (2)	0.0058 (19)	0.0081 (17)	0.0047 (19)
N1	0.059 (2)	0.052 (2)	0.0225 (18)	−0.0017 (19)	0.0104 (16)	0.0012 (16)
C2	0.054 (3)	0.057 (3)	0.037 (3)	0.001 (2)	0.005 (2)	−0.006 (2)
N2	0.062 (2)	0.043 (2)	0.0248 (18)	0.0046 (18)	0.0097 (16)	−0.0007 (16)
C3	0.052 (3)	0.078 (4)	0.031 (2)	0.012 (3)	−0.002 (2)	−0.013 (3)
C4	0.064 (3)	0.079 (4)	0.024 (2)	0.019 (3)	0.007 (2)	0.003 (2)
C5	0.071 (3)	0.051 (3)	0.034 (3)	0.013 (2)	0.016 (2)	0.008 (2)
C6	0.046 (2)	0.053 (3)	0.031 (2)	0.009 (2)	0.0105 (18)	0.002 (2)
C7	0.0324 (19)	0.060 (3)	0.0186 (19)	0.013 (2)	0.0016 (15)	−0.0124 (19)
C8	0.049 (2)	0.041 (2)	0.024 (2)	0.007 (2)	0.0071 (17)	−0.0004 (18)
C9	0.042 (2)	0.036 (2)	0.026 (2)	0.0013 (18)	0.0036 (17)	−0.0014 (18)
C10	0.057 (3)	0.046 (3)	0.036 (2)	0.005 (2)	0.005 (2)	−0.007 (2)
C11	0.047 (2)	0.058 (3)	0.029 (2)	0.012 (2)	0.0056 (19)	−0.011 (2)

### Geometric parameters (Å, º)

**Table:**

S—C8	1.723 (5)	C3—C4	1.363 (7)
S—C1	1.723 (4)	C3—H3B	0.9300
O2—C11	1.232 (5)	C4—C5	1.382 (7)
O1—C9	1.333 (5)	C4—H4A	0.9300
O1—C10	1.435 (5)	C5—C6	1.418 (6)
C1—C6	1.390 (6)	C5—H5A	0.9300
C1—C2	1.406 (6)	C6—C7	1.485 (6)
N1—C11	1.323 (6)	C7—C8	1.438 (6)
N1—N2	1.387 (5)	C7—H7A	0.9300
N1—H1A	0.8600	C8—C9	1.477 (5)
C2—C3	1.377 (6)	C10—C11	1.492 (6)
C2—H2B	0.9300	C10—H10A	0.9700
N2—C9	1.274 (5)	C10—H10B	0.9700
			
C8—S—C1	90.0 (2)	C1—C6—C5	118.9 (4)
C9—O1—C10	118.7 (3)	C1—C6—C7	115.1 (4)
C6—C1—C2	121.8 (4)	C5—C6—C7	125.9 (4)
C6—C1—S	113.0 (3)	C8—C7—C6	104.4 (4)
C2—C1—S	125.2 (4)	C8—C7—H7A	127.8
C11—N1—N2	125.5 (4)	C6—C7—H7A	127.8
C11—N1—H1A	117.3	C7—C8—C9	124.0 (4)
N2—N1—H1A	117.3	C7—C8—S	117.5 (3)
C3—C2—C1	117.8 (5)	C9—C8—S	118.6 (3)
C3—C2—H2B	121.1	N2—C9—O1	128.1 (4)
C1—C2—H2B	121.1	N2—C9—C8	118.8 (4)
C9—N2—N1	115.5 (4)	O1—C9—C8	113.0 (3)
C4—C3—C2	121.1 (5)	O1—C10—C11	114.6 (4)
C4—C3—H3B	119.5	O1—C10—H10A	108.6
C2—C3—H3B	119.5	C11—C10—H10A	108.6
C3—C4—C5	122.5 (4)	O1—C10—H10B	108.6
C3—C4—H4A	118.8	C11—C10—H10B	108.6
C5—C4—H4A	118.8	H10A—C10—H10B	107.6
C4—C5—C6	117.9 (5)	O2—C11—N1	123.6 (4)
C4—C5—H5A	121.0	O2—C11—C10	119.0 (4)
C6—C5—H5A	121.0	N1—C11—C10	117.4 (4)
			
C8—S—C1—C6	1.8 (3)	C6—C7—C8—S	0.0 (4)
C8—S—C1—C2	−179.4 (4)	C1—S—C8—C7	−1.0 (3)
C6—C1—C2—C3	−0.9 (6)	C1—S—C8—C9	177.3 (3)
S—C1—C2—C3	−179.7 (3)	N1—N2—C9—O1	−1.6 (6)
C11—N1—N2—C9	1.8 (6)	N1—N2—C9—C8	−178.0 (3)
C1—C2—C3—C4	1.7 (7)	C10—O1—C9—N2	2.6 (7)
C2—C3—C4—C5	−2.2 (7)	C10—O1—C9—C8	179.1 (4)
C3—C4—C5—C6	1.9 (7)	C7—C8—C9—N2	176.5 (4)
C2—C1—C6—C5	0.7 (6)	S—C8—C9—N2	−1.7 (5)
S—C1—C6—C5	179.5 (3)	C7—C8—C9—O1	−0.4 (6)
C2—C1—C6—C7	178.9 (4)	S—C8—C9—O1	−178.6 (3)
S—C1—C6—C7	−2.2 (4)	C9—O1—C10—C11	−3.3 (6)
C4—C5—C6—C1	−1.1 (6)	N2—N1—C11—O2	177.0 (4)
C4—C5—C6—C7	−179.1 (4)	N2—N1—C11—C10	−2.8 (6)
C1—C6—C7—C8	1.4 (4)	O1—C10—C11—O2	−176.5 (4)
C5—C6—C7—C8	179.5 (4)	O1—C10—C11—N1	3.4 (6)
C6—C7—C8—C9	−178.2 (4)		

### Hydrogen-bond geometry (Å, º)

**Table:**

*D*—H···*A*	*D*—H	H···*A*	*D*···*A*	*D*—H···*A*
N1—H1*A*···O2^i^	0.86	2.03	2.848 (5)	158

Symmetry code: (i) −*x*+3/2, *y*−1/2, −*z*+5/2.
